# Comorbid asthma in patients with chronic rhinosinusitis with nasal polyps: did dupilumab make a difference?

**DOI:** 10.1186/s12890-023-02556-8

**Published:** 2023-07-18

**Authors:** Mona Al-Ahmad, Asmaa Ali, Mustafa Khalaf, Abdulmohsen Alterki, Tito Rodriguez-Bouza

**Affiliations:** 1 Department of Microbiology, College of Medicine, Kuwait University, Kuwait City, Kuwait; 2 Department of Allergy, Al-Rashed Allergy Center, Ministry of Health, Kuwait City, Kuwait; 3 Department of Laboratory Medicine, School of Medicine, Jiangsu University, Zhenjiang, China; 4 Department of Pulmonary Medicine, Abbassia Chest Hospital, Ministry of Health, Cairo, Egypt; 5 Department of Otolaryngology, Head and Neck Surgery, Zain and Al Sabah Hospital, Ministry of Health, Kuwait City, Kuwait; 6 Clinica la Maestranza, Madrid, Spain

**Keywords:** Chronic rhinosinusitis, Nasal polyps, Asthma, Dupilumab, Sinonasal outcome test, Asthma control test

## Abstract

**Background:**

The clinical heterogeneity of chronic rhinosinusitis (CRS) and bronchial asthma is attributable to different underlying inflammatory profiles. However, the similarity between CRS with nasal polyps (CRSwNP) and type-2 asthma pathophysiology speculates that one biological therapy could affect both comorbidities. Despite dupilumab, a monoclonal antibody that targets IL-4α and IL-13 receptors, being used in patients with nasal polyps and severe asthma, real-life data about its efficacy in improving the quality of life and patient symptoms is still lacking. This study’s primary objective was to evaluate dupilumab treatment’s effect on the frequency of olfactory symptoms and health-related quality of life tests as measured by the Sino-nasal outcome test (SNOT-22) in patients with NP. The secondary objective was the effect of dupilumab on asthma symptom control as measured by the asthma control test (ACT).

**Methods:**

A prospective study was conducted of 166 patients with CRSwNP, with or without asthma. The following variables were collected at baseline and after at least six months of continuous dupilumab therapy; SNOT-22, olfactory symptoms frequency, and ACT score.

**Results:**

Asthma prevalence in patients with CRSwNP was high (59.63%), and being female with a history of frequent use of oral corticosteroid (OCS) courses and repeated unsuccessful nasal and para-nasal surgeries for polyposis increased the likelihood of having underlying asthma by 2, 1 and 4 times more, respectively. Additionally, being asthmatic required a longer duration of dupilumab treatment. However, both the health-related quality of life and olfactory symptoms improved equally in both groups.

**Conclusion:**

Even with associated comorbid asthma in patients with CRSwNP, treatment with dupilumab could improve the quality of life, olfactory symptoms, and asthma symptom control.

## Introduction

Affecting 5–28% of the general population, chronic rhinosinusitis (CRS) is a prevalent condition worldwide that places a heavy financial burden due to increased healthcare costs and loss of productivity [1–5]. CRS is a group of disorders that cause inflammation of the sinonasal mucosa and have a multifactorial etiology involving immunological and epithelial barrier components influenced by the microbiota, environment, and genetic factors [6]. Along with objective evidence, at least two out of the following four cardinal symptoms are necessary to diagnose CRS: facial pain/pressure, hyposmia/anosmia, nasal discharge, and nasal obstruction for at least 12 weeks in a row. Physical examination (anterior rhinoscopy, endoscopy) or radiography, ideally from sinus computed tomography, can provide objective evidence of CRS [7].

Two significant phenotypes are defined for CRS based on nasal endoscopic findings: CRS without nasal polyps (CRSsNP) and CRS with nasal polyps (CRSwNP). CRSwNP, which comprises about 18–20% of all CRS cases, is linked to significant morbidity and may impact adults’ lower airway disease status [8, 9]. Moreover, CRSwNP and asthma are closely linked, as implied by epidemiological, clinical, and pathophysiological studies [10, 11]. Correlation between the inflammatory profiles of nasal and bronchial biopsies in patients with CRSwNP is significant (*P* < 0.01),[12] which is reflected in the direct relationship between the inflammation in the nasal mucosa and lower airways and adding support to the united airways concept already described in patients with asthma and comorbid allergic rhinitis [6, 13–15]. From a pathophysiological view, both disorders, CRSwNP, and asthma share the same type 2 immunopathology (involvement of IgE, eosinophils, interleukin-4 (IL-4)/IL-13, and IL-5) and exhibit epithelial barrier dysfunction [16–21].

Patients with CRSwNP and comorbid asthma demonstrate IgE-mediated release of immune mediators and upregulation of type 2 cytokines (IL-4, IL5, and IL-13) in the upper and lower airways [20, 21]. Further, tissue eosinophilia and high local IgE levels are typically characterized in CRSwNP with asthma [6]. Higher rates of polyp recurrence and difficult-to-treat asthma are often demonstrated in patients with CRSwNP identified as having a type-2 immune response [22]. The association between olfactory dysfunction in CRSwNP and tissue eosinophilia implies a role for eosinophils (or their associated cytokines) in partial or total loss of smell typically manifest in CRS [23, 24].

Dupilumab, an IL-4 receptor alpha antagonist, is a human monoclonal antibody of the immunoglobulin G4 subclass that specifically binds to the IL-4 receptor alpha subunit, which is shared by the IL-4 and IL-13 receptor complexes, thus inhibits IL-4 and IL-13 signaling. Dupilumab inhibits IL-4 signaling via the type 1 receptor and both IL-4 and IL-13 signaling via the type 2 receptor. It inhibits IL-4 and IL-13 cytokine-induced responses by blocking the IL-4R alpha subunit, including releasing proinflammatory cytokines, chemokines, and immunoglobulin E [25]. By blocking the shared receptor component for IL-4 and IL-13, dupilumab improves upper and lower airway outcome measures in patients with severe CRSwNP and comorbid asthma, as reported by the body of evidence [26–30]. The present study evaluated dupilumab efficacy in improving CRSwNP patients, with or without asthma, regarding the quality of life, olfactory symptoms, and asthma symptom control.

## Patients and methods

### Study design, patients, and outcome

This prospective follow-up study was conducted in a tertiary allergy center in Kuwait, Al-Rashed Allergy Center, from January 2021 till the end of May 2022. The primary outcome was to evaluate the frequency of olfactory symptoms (anosmia and hyposmia) and measure SNOT-22 after at least six months of continuous therapy with Dupilumab. The secondary outcome measured the changes in ACT scores in patients with asthma.

Diagnosis of patients with CRSwNP was based on clinical diagnostic criteria; all patients had an endoscopic assessment for nasal polyps; additionally, 141 of 166 (84.9%) patients had a history of nasal polypectomy where the radiological finding of all cases showed polypoidal nasal mucosal thickness. Individuals displaying symptoms indicative of asthma were diagnosed based on the variability of their symptoms and the reversibility of their FEV1%. If the initial spirometry results were inconclusive, further tests such as PEF variability and FeNO were conducted to confirm the diagnosis [31]. Individuals with moderate to severe chronic rhinosinusitis with nasal polyps, with or without asthma, were assessed for their eligibility to start biological therapy as per the guidelines of EPOS, 2020 [32]. Those who received dupilumab treatment for at least six months were enrolled in the study.

Demographic data, SNOT-22, and ACT scores were collected from patients’ medical charts, and all patients were followed up for at least three months. SNOT-22, olfactory symptoms frequency and ACT were re-measured. As per *Toma et al.*, the SNOT-22 score was classified into mild, moderate, and severe [33], which later was adapted as mild and moderate versus severe.

### Ethics approval and consent to participate

#### Ethical approval

has been obtained from Kuwait University and the Ministry of Health, in accordance with the Helsinki Declaration protocol (Research study number 2121/2022), to ensure that the research is conducted ethically and in compliance with internationally recognized standards. Informed consent has been obtained from all participants involved in the study, as well as their legal guardians, to ensure that they are fully aware of the nature and purpose of the research. They have given their voluntary and informed consent to participate.

### Data collection

Basic demographic data were collected from the patient’s files, including age, sex, SNOT-22 score, nasal polyps surgery, oral corticosteroid use, presence of anosmia/hyposmia, ACT, and duration of dupilumab therapy. After at least three months of patient recruitment, ACT score, SNOT-22 score, and olfactory symptoms were reassessed.

### Statistical methods

Categorical variables were presented as numbers and percentages, while continuous variables were provided as mean and standard deviation (SD). The normality of data was examined using the Shapiro-Wilk test, and the descriptive statistics were performed using Minitab 17.1.0.0 for Windows (Minitab Inc., 2013, Pennsylvania, USA). The comparison between two means or medians was made using an independent t-test or Mann-Whitney test, while the frequency comparison was made using the chi-square test. We conducted a multiple logistic regression analysis using a stepwise elimination technique to identify potential predictors of underlying asthma in patients with CRS and nasal polyps, in which the collinearity among the potential confounders was estimated using correlation coefficient, and a sensitivity analysis was done within the test by removing one potential confounder at a time and examining the changes in the coefficients of the independent variable(s). The performance of oral corticosteroid course number in predicting associated asthma was evaluated using the ROC curve, and the area under the curve (AUC) above 0.6 was considered acceptable. All tests were two-sided, and *a p-value* below 0.05 was considered significant.

## Results

### Basic characteristics of patients with CRSwNP

**Fig. 1:**
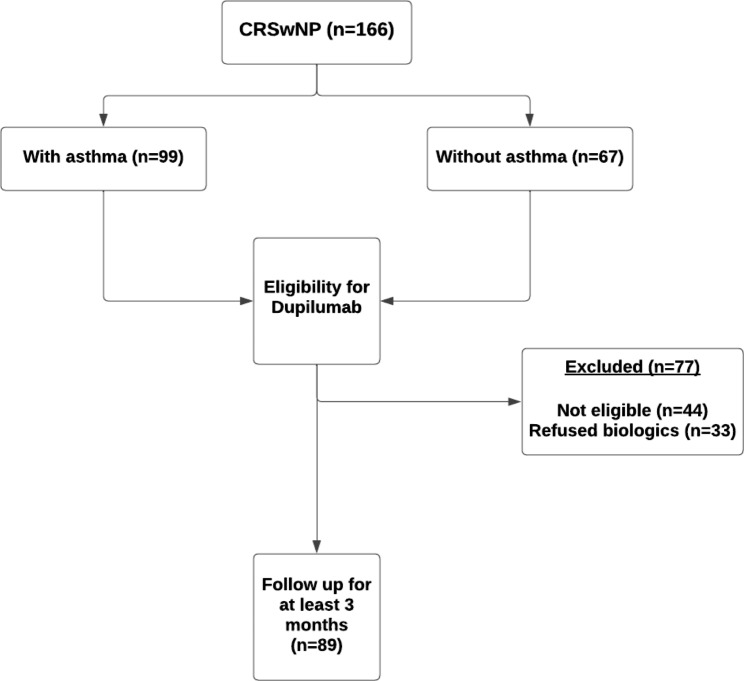
Recruitment for biological therapy

A total of 166 patients were diagnosed with CRSwNP, of which 99 cases (59.63%) were also diagnosed with asthma. However, 25 patients were excluded from the study due to not meeting the criteria for starting biologics, and an additional 33 individuals opted out of biologic treatment, as shown in Fig. 1. The mean (SD) age of patients was 43 (12) years, and 49.4% were females (Table 1); SNOT-22 score, polyposis surgery, oral corticosteroid use, and anosmia/hyposmia were significantly higher in patients with CRSwNP and asthma compared to others without asthma.

**Table 1:** Basic characteristics of patients with CRSwNP

Factors	Total (n = 166)	CRSwNP without asthma(n = 67)	CRSwNP with asthma (n = 99)	*P*
Age(mean ± SD)	43.295 ± 12.716	43.3 ± 12	43.3 ± 13.2	0.98
Female sex (n, %)	82 (49.4)	27 (40.3)	55 (55.56)	**0.05**ƪ
SNOT-22 score(mean ± SD)	58.73 ± 24.16	53 ± 25.4	62.6 ± 22.6	**0.01** ^**†**^
SNOT-22 (severe)(n, %)	104 (62.65)	36 (53.73)	68 (68.69)	**0.05**ƪ
PS (Yes)(n, %)	141 (84.94)	50 (74.63)	91 (91.92)	**0.002**ƪ
PSN(median, IQR)	2(1–3)	2(0–3)	2(1–3)	**0.03** ^**††**^
OCS-use (Yes)(n, %)	102 (61.45)	36 (53.73)	66 (66.67)	**0.05**ƪ
OCS-CN(median, IQR)	1(0–3)	1(0–2)	1(0–3)	**0.01** ^**††**^
Anosmia/hyposmia (Yes) (n, %)	159 (95.78)	66 (98.51)	93 (93.94)	0.15
ACT (mean ± SD)	-----	-----	16.923 ± 5.826	-----

The continuous data represented as mean and stander deviation or median and inter quartile range, and categorical data as number and percentage, SD: stander deviation, N: number, IQR: inter quartile range, SNOT: Sinonasal Outcome Test, PS: Polyposis surgery, PSN: polyposis surgery number, OCS: oral corticosteroid, OCS-CN: oral corticosteroid courses number, ACT: asthma control test, ƪ: Chi square test, †: independent t-test, ††: Mann Whitney test, P-value < 0.05 considered significant

### Asthma risk factors

**Fig. 2:**
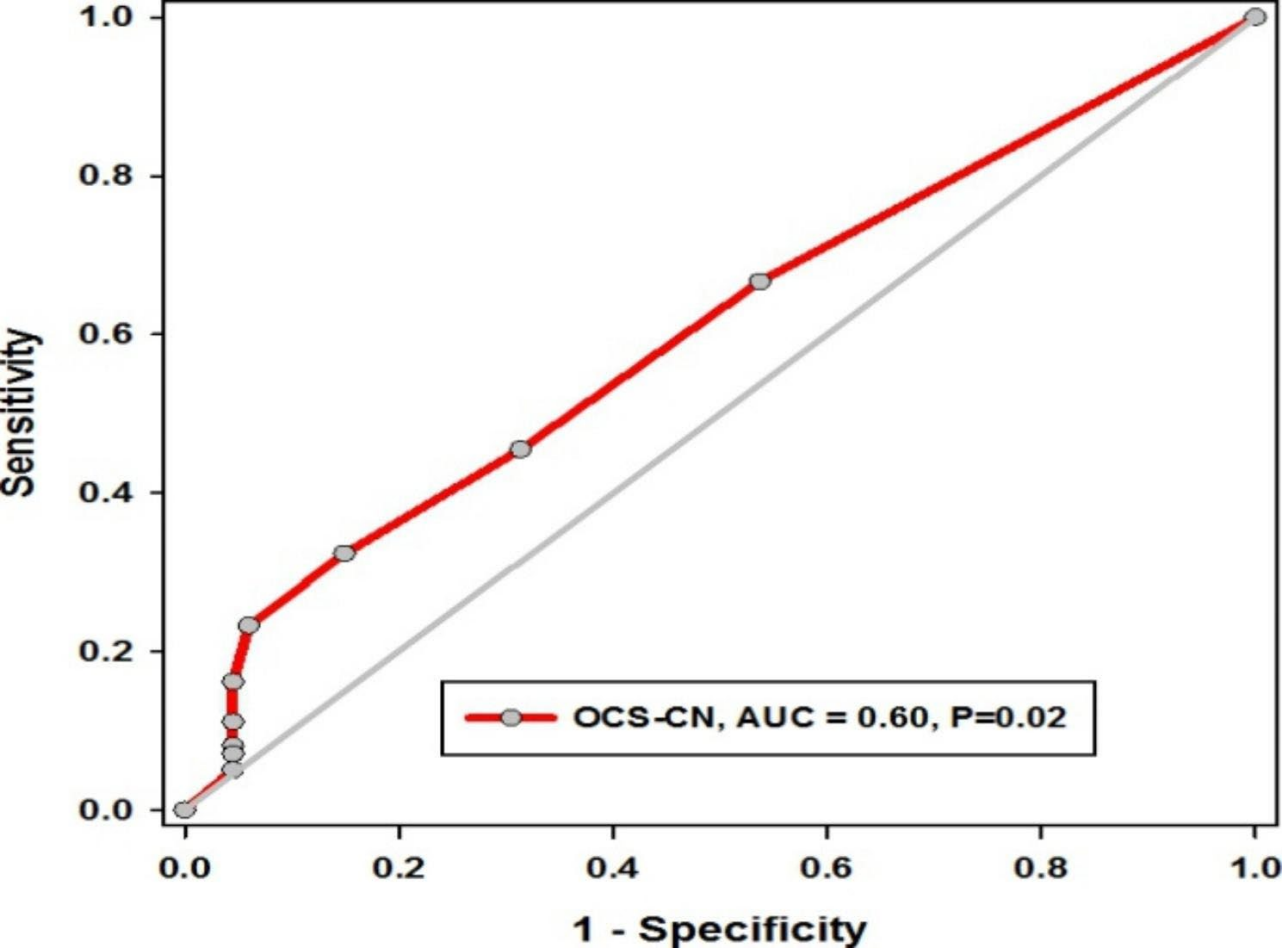
ROC curve of oral corticosteroid course number to discriminate asthma coexistence in patients with CRSwNP OCS-CN: oral corticosteroid course number, AUC: area under the curve, *P* < 0.05 considered significant: Receiver operating characteristic curve (ROC-curve) showed good utility of OCS-CN, AUC = 0.6, *P* = 0.02

In patients with CRSwNP, female sex and polyposis surgery were found to be significant independent risk factors for asthma, with a p-value of less than 0.05 and odds ratios of 2.13 and 4.04, respectively. Additionally, every additional course of OCS increased the likelihood of asthma coexistence up to two times more, with an odds ratio of 1.56 and a p-value of 0.05 (Table 2). Furthermore, ROC analysis was conducted to determine the ability of the number of oral corticosteroid courses to discriminate asthma coexistence in patients with CRSwNP. The results showed an area under the curve (AUC) of 0.60 with a p-value of 0.02 (Fig. 2). When the cutoff number was set at > 1.5, 2.5, and 3.5, the sensitivity and specificity were as follows: 67% and 69%, 45% and 85%, and 23% and 94%, respectively (Table 3).

**Table 2:** Asthma independent risk factors in patients with CRSwNP

Factors	OR	95% CI	*P*
Sex (female)	2.13	(1.0874,4.1688)	**0.025**
OCS-CN	1.56	(0.9849,1.2801)	**0.053**
PS	4.04	(1.5467,10.5767)	**0.003**

Multiple logistic regressions with stepwise elimination methods, The test of fitness: Hosmer-Lemeshow, X^2^ = 9.1, P = 0.31, OR: odd ratio, CI: confidence interval, *P* < 0.05 considered significant

**Table 3:** Predictive utility of oral corticosteroid courses number for asthma coexistence in patients with CRSwNP

Cutoff >	Sensitivity	95% CI	Specificity	95% CI	PV +	PV -
1.5	67%	0.5648 to 0.7582	69%	0.5616 to 0.7944	24%	85%
2.5	45%	0.3541 to 0.5577	85%	0.7426 to 0.9260	32%	85%
3.5	23%	0.1533 to 0.3279	94%	0.8541 to 0.9835	46%	85%

CI: confidence interval, PPV: positive predictive value, NPV: negative predictive value

### Impact of dupilumab treatment

**Fig. 3:**
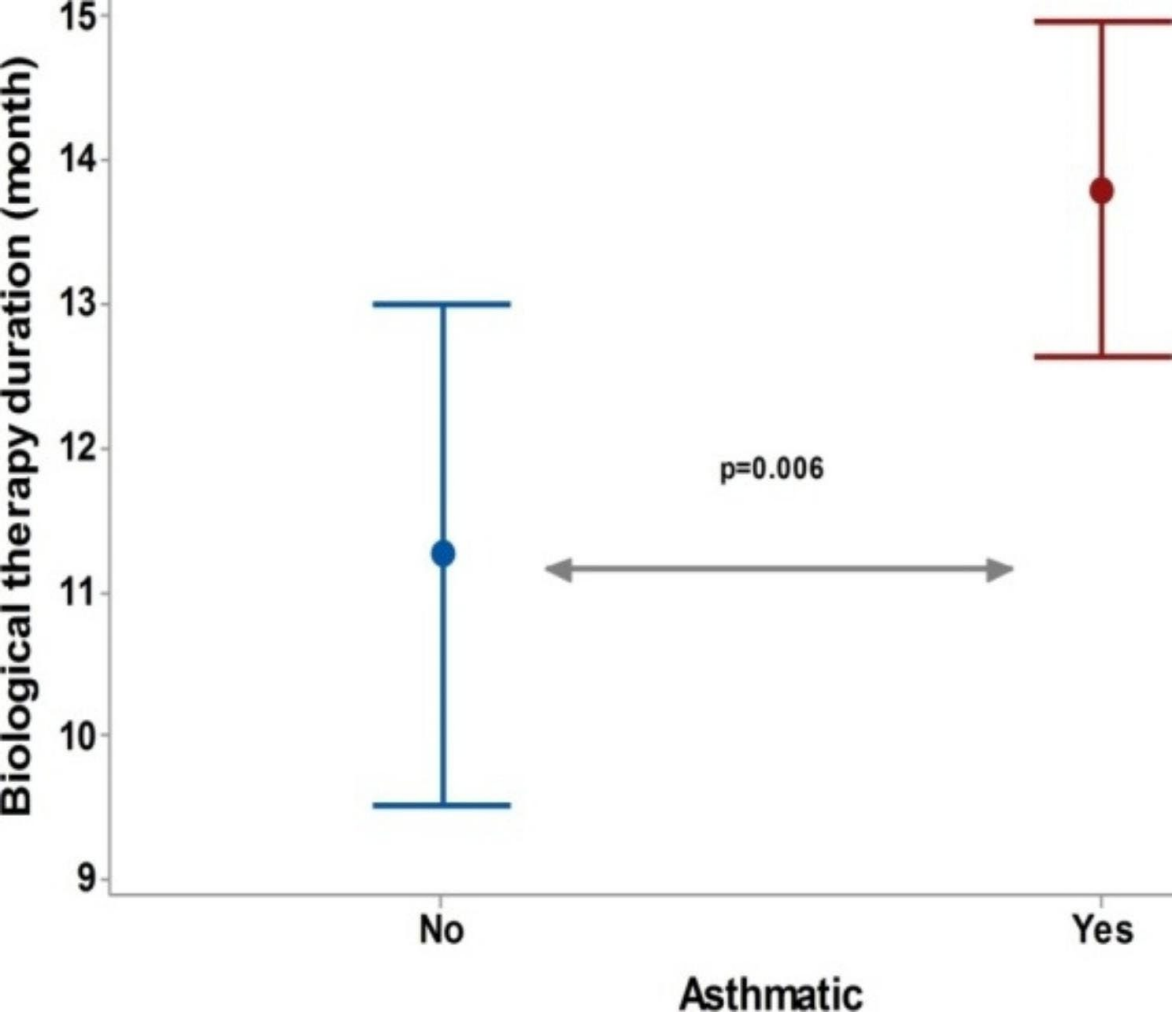
Duration of Dupilumab in CRSwNP patients with and without asthma In non asthmatic patients the (mean ± SD) duration of dupilumab was (11.2 ± 4.1) month versus (13.8 ± 4.4) month in asthmatic group, the test of significant: Independent t-test, *P* < 0.05 considered significant

**Fig. 4:**
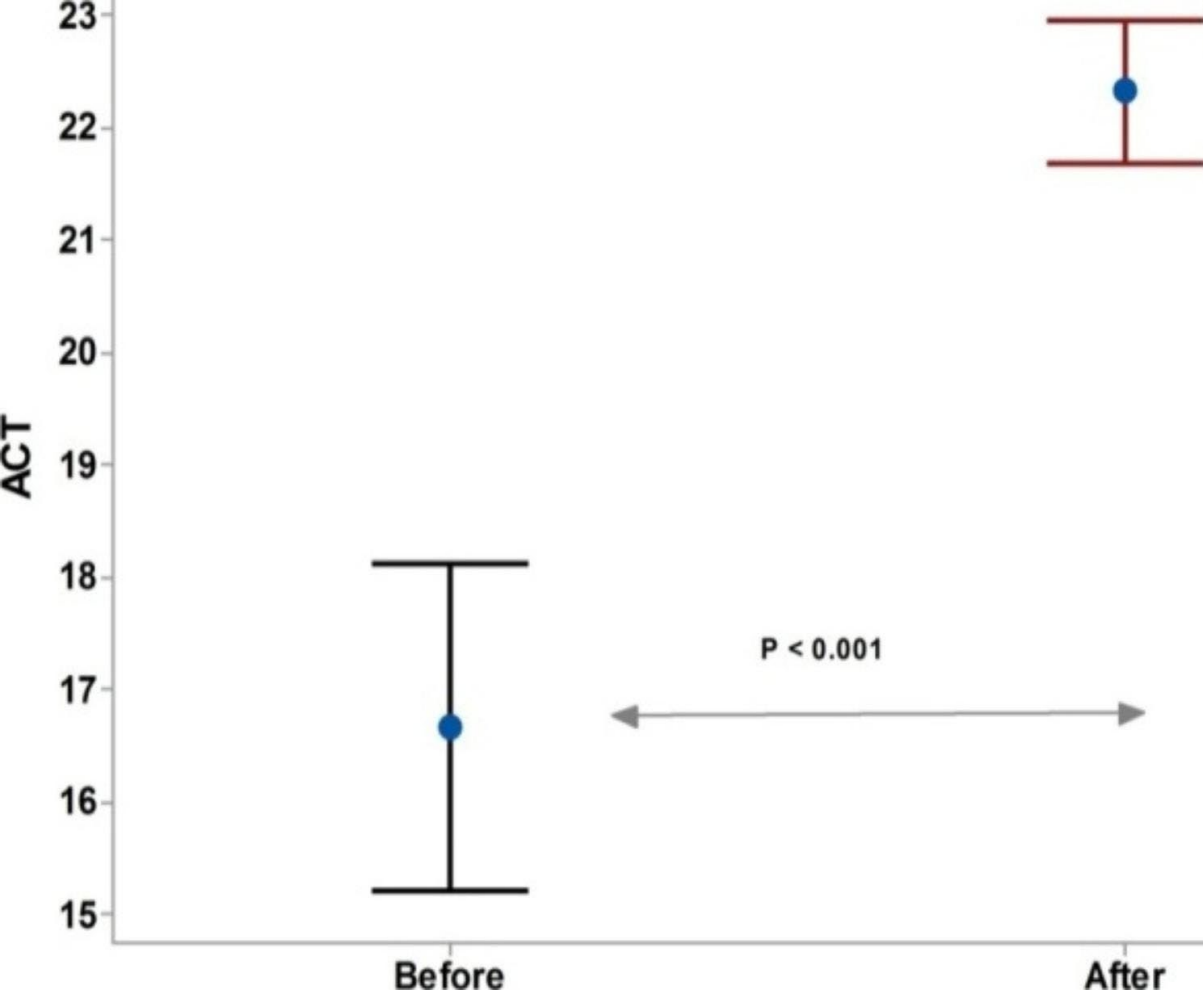
Asthma control test score after Dupilumab Test of significant: independent t-test, *P* < 0.05 considered significant

**Fig. 5:**
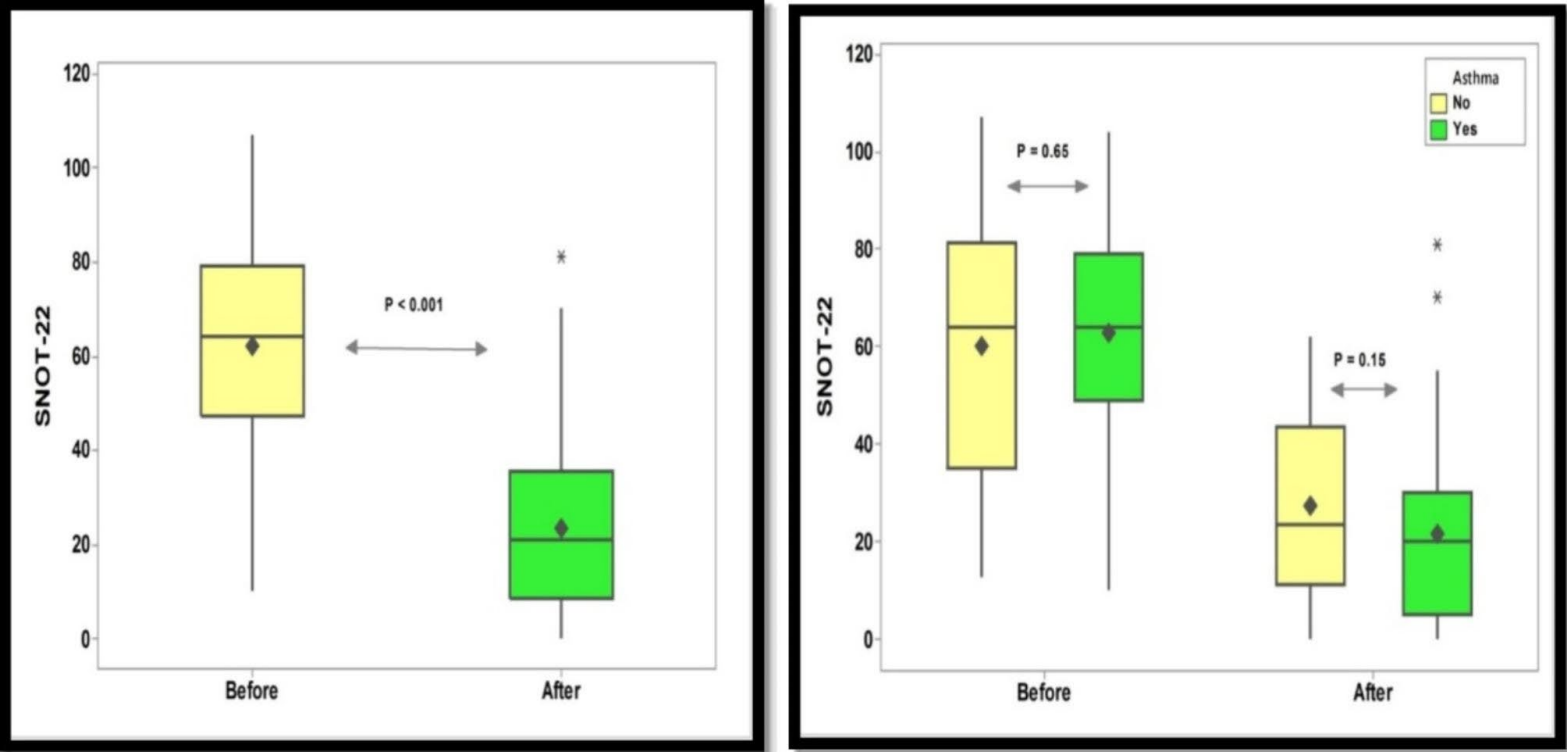
Impact of Dupilumab treatment on SNOT-22 score SNOT-22: Sino-Nasal outcome Test, Test of significant: independent t-test, *P* < 0.05 considered significant

The duration of dupilumab in CRSwNP patients with asthma was significantly higher than in those without asthma (Fig. 3). The ACT scores after dupilumab treatment was significantly higher than before dupilumab, Fig. 4. For the SNOT-22 score, a significant decrease was recorded after the dupilumab treatment compared to the baseline. However, no significant difference in the SNOT-22 scores before and after dupilumab among each subgroup of CRSwNP patients, Fig. 5.

**Fig. 6:**
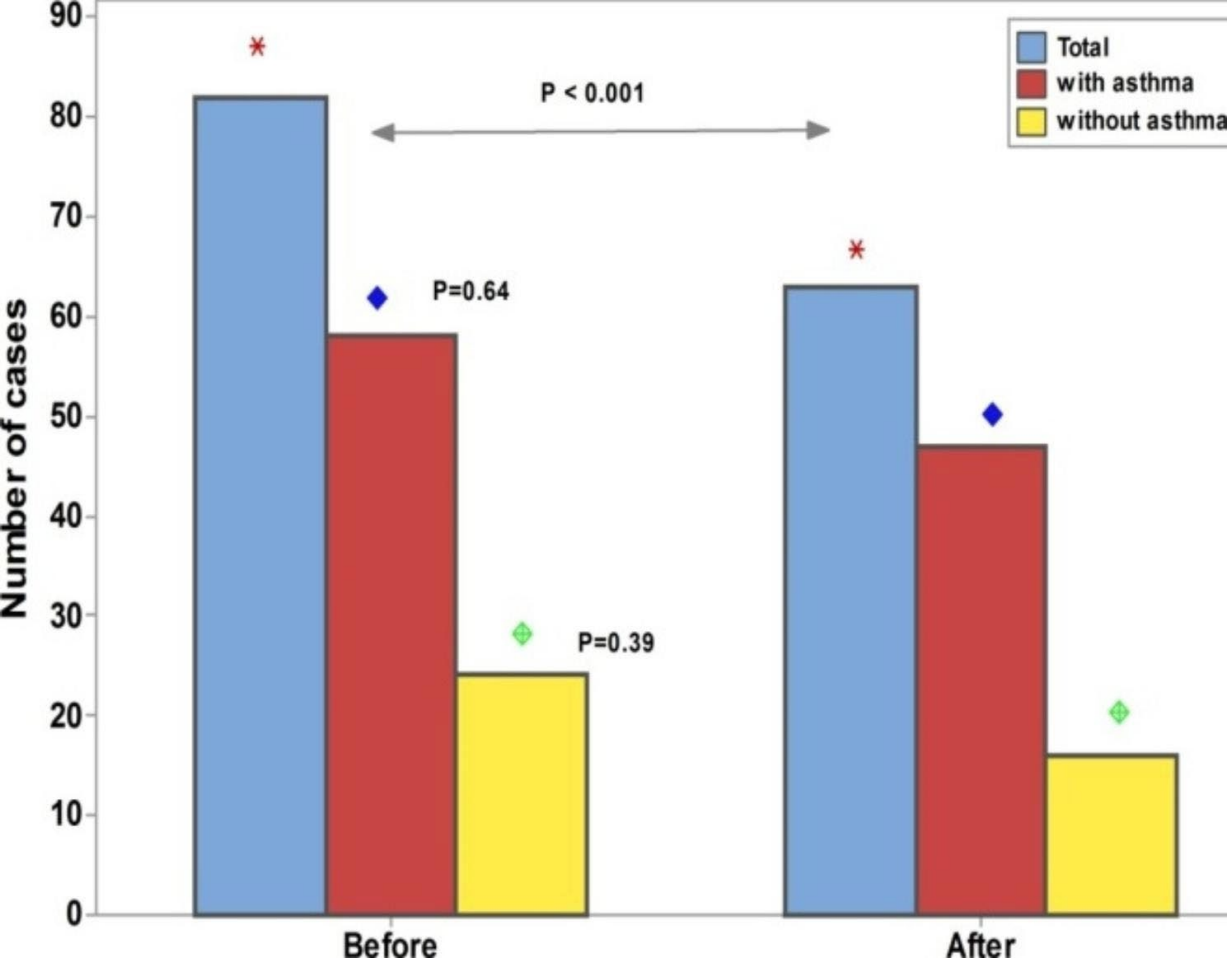
Impact of Dupilumab on olfactory symptoms The test of significant: Chi square test, *P* < 0.05 considered significant

The impact of dupilumab on olfactory symptoms in Fig. 6; a significant decrease in the overall frequency of anosmia after dupilumab treatment; however, the effect of the presence of asthma was insignificant.

## Discussion

This analysis examines real-life data on the effectiveness of dupilumab in improving the quality of life and olfactory symptoms of patients with CRSwNP and associated asthma. Real-world studies are conducted to verify, expand upon, and complement the findings of RCTs to comprehend better how efficacy data are applied at the point of care in routine clinical practice. Of the 166 evaluated patients, 99 (59.6%) were diagnosed with CRSwNP with asthma. These figures were comparable to others reported by *Håkansson et al.* [34]; to investigate the association between CRSwNP and asthma; they conducted a prospective clinical trial involving 40 CRS patients scheduled for functional endoscopic sinus surgery (FESS) and 21 control subjects. 25% (25%) of the CRSwNP patients had undiagnosed asthma, while asthma was diagnosed in 65% of them. Furthermore, lower airway obstruction in people with asthma and chronic bronchitis was linked to CRSwNP. Although asthma is frequently misdiagnosed, the fact that 25% of CRSwNP patients had undiagnosed and untreated asthma was unexpected [35]. This necessitates more collaboration between otorhinolaryngologists and pulmonologists.

Female sex, SNOT-22 score, SNOT-22 (severe), polyposis surgery, polyposis surgery number, OCS use, and OCS course number; all were statistically significantly higher in the subgroup of patients diagnosed with CRSwNP and asthma compared to the other one. These results imply that asthma independently influences CRSwNP patients’ quality of life.

The results of the current study revealed that female sex and polyposis surgery are independent risk factors for asthma in patients with CRSwNP (P < 0.05). These are compatible with a previous cross-sectional study by *Li* and colleagues; based on medical histories and clinical examinations, risk factors for concomitant asthma in CRSwNP patients were investigated. The study showed that among patients with CRSwNP, having a female gender, a history of allergic rhinitis, elevated serum T-IgE levels (> 69.0 kU/L), and a blood eosinophils count (> 0.35 109/L) were independent risk factors for comorbid asthma [36]. Female gender has been linked to an increased incidence of asthma in previous studies, and the presence of this factor may make asthma even more likely to occur. Numerous epidemiological studies indicate that women are more likely than men to have adult-onset asthma and more severe conditions [37–39].

Exploring risk factors linked to a specific phenotype may be more beneficial for managing individuals with asthma. Compared to asthmatics without CRSwNP, those with concomitant CRSwNP are likely to have more severe and persistent asthma, a lower quality of life, and fixed airflow limitation [26]. Also, asthma is more difficult to manage and more likely to exacerbate when nasal polyposis occurs. It has been demonstrated that nasal polyposis predicts the chronicity and durability of new-onset asthma [40, 41]. The emergence of asthma in CRSwNP patients appears to be cryptogenic; nevertheless, many individuals go misdiagnosed, and some may be asymptomatic [26, 42, 43]. Even rhinologists sometimes mistakenly diagnose CRSwNP as the cause of coughing and dyspnea rather than asthma. As a result, concomitant asthma in CRSwNP patients may be overlooked, especially at an early stage [26, 34, 44]. In order to develop a realistic strategy for specifically rhinologists to screen individuals at high risk, it is vital to investigate the risk factors associated with comorbid asthma in patients with CRSwNP [36].

Our results revealed that the ACT score was significantly higher after dupilumab treatment than before dupilumab treatment, which follows data reported by *Dupin et al.* [45]. They reported a significant improvement in median ACT scores from 14 (7–16) to 22 (17–24) (P < 0.001) after 12 months of dupilumab treatment in patients with severe asthma. These data are in accordance with results reported by *Mümmler* and colleagues [46]. They retrospectively analyzed 38 patients who had previously received anti-IgE or anti-IL-5/IL-5 receptor medication and were switched to dupilumab due to a bad outcome. The investigators revealed that the ACT score increased by a mean of 2.9 (P < 0.0001) while exacerbations decreased significantly (P < 0.0001). Another supportive result came from *Pelaia et al.* [47]; following four weeks with dupilumab, all patients observed significant improvement in severe asthma and nasal polyposis; the ACT score had significantly increased (*p* < 0.0001); moreover, in a more comprehensive observational study, the ACT score increased from 14 (10–18) to 22 (20–24) (p < 0.0001), [48].

For the SNOT-22 score, the significant decrease recorded after the dupilumab treatment compared to the baseline score in the current study indicated a significant impact on the patient’s QoL. This result aligns with data from a randomized controlled trial by *Mustafa* and colleagues [49]. AERD patients 18 or older and with a SNOT 22 score below 19 were eligible for their study. The study aimed to assess dupilumab’s effectiveness in treating AERD patients with uncontrolled CRSwNP. Their findings showed that following six months of dupilumab medication, the median baseline SNOT 22 score significantly improved from 46 [IQR: 34 to 64.8] to 9.5 [IQR: 2.5 to 19](P = 0.0050).

For the olfactory symptoms, we reported a significant decrease in the overall frequency of anosmia after dupilumab treatment which is consistent with the results demonstrated by *Buchheit* and colleagues [50]. They include 22 AERD patients treated with dupilumab for three months for severe asthma and CRS with nasal polyps. Clinical outcomes were assessed at baseline, 1 and 3 months after initiation of dupilumab. The study participants showed rapid improvement in clinical measures, including the sense of smell, sinonasal symptoms, and lung function after one month of treatment with dupilumab; moreover, the improvements were sustained after three months of dupilumab. There were no changes in nasal eosinophilia after dupilumab. The authors concluded that the therapeutic effects of dupilumab are likely due to decreased IL-4Rα signaling on respiratory tissue granulocytes, epithelial cells, and B cells. More than 3400 individuals with CRSwNP from 29 randomized trials evaluating nine treatment options were included in a systematic review and network meta-analysis. The findings showed that there is moderate to high certainty evidence that dupilumab improves HRQoL, sinusitis symptoms, smell, rescue OCS, rescue nasal polyp surgery, and nasal polyp size compared to standard therapy [51].

## Conclusion

More than half of our cohort with CRSwNP had associated asthma. However, treatment with Dupilumab effectively controlled asthma symptoms and improved both the quality of life and olfactory symptoms.

## Data Availability

The datasets used during the current study available from the corresponding author on reasonable request.
